# Diagnostic efficacy of absolute and relative myocardial blood flow of stress dynamic CT myocardial perfusion for detecting myocardial ischemia in patients with hemodynamically significant coronary artery disease

**DOI:** 10.3389/fcvm.2024.1398635

**Published:** 2024-07-12

**Authors:** Weifang Kong, Bingzhu Long, Hongyun Huang, Fang Li, Yuefeng He, Xinyue Chen, Hong Pu, Guojin Zhang, Lan Shang

**Affiliations:** ^1^ Department of Radiology, Sichuan Provincial People’s Hospital, School of Medicine, University of Electronic Science and Technology of China, Chengdu, China; ^2^ School of Medicine, University of Electronic Science and Technology of China, Chengdu, China; ^3^ Department of Diagnostic Imaging, CT Collaboration, Siemens Healthineers, Chengdu, China

**Keywords:** myocardial ischemia, coronary artery disease, relative MBF, absolute MBF, hemodynamically significant CAD, computed tomography perfusion

## Abstract

**Introduction:**

Stress dynamic computed tomography myocardial perfusion imaging (CT-MPI) is an accurate quantitative method for diagnosing myocardial ischemia in coronary artery disease (CAD). However, its clinical application has been limited, partly due to the varied cutoff values for absolute myocardial blood flow (MBFa) and the uncertain value of the relative myocardial blood flow ratio (MBF-ratio). This study aimed to compare the diagnostic efficacy of and investigate the optimal cutoff values for MBFa and the MBF-ratio in CT-MPI for diagnosing myocardial ischemia in patients with hemodynamically significant CAD.

**Methods:**

Patients with suspected or known hemodynamically significant CAD who underwent CT-MPI + CT angiography and invasive coronary angiography (ICA)/fractional flow reserve (FFR) between October 2020 and December 2023 were retrospectively evaluated. ICA ≥80% or FFR ≤0.8 were set as the diagnostic standards for functional ischemia. The patients and vessels were categorized into ischemic and non-ischemic groups, and differences in MBFa and the MBF-ratio were compared between the groups. The area under the curve (AUC) and optimal cutoff values were calculated. Diagnostic efficacy parameters, such as sensitivity, specificity, and accuracy, were also compared. In addition, a consistency test was performed.

**Results:**

A total of 46 patients (mean age: 65.37 ± 8.25 years; 120 vessels) were evaluated. Hemodynamically significant stenosis was detected in 30/46 patients (48%) and 81/120 vessels (67.5%). The MBFa and MBF-ratio values were significantly lower in the ischemic than in the non-ischemic group; in the per-vessel analysis, the MBFa values were 73 vs. 128 (*P *< 0.001) and the MBF-ratio values were 0.781 vs. 0.856 (*P *< 0.001), respectively. The optimal cutoff values for MBFa and the MBF-ratio were 117.71 and 0.67, respectively. MBFa demonstrated a sensitivity, specificity, accuracy, AUC, positive predictive value, negative predictive value, and kappa value of 97.44%, 74.07%, 81.66%, 0.936 [95% confidence interval (CI): 0.876–0.973, *P *< 0.001], 63.33%, 98.36%, and 0.631 (95% CI: 0.500–0.762), respectively. The corresponding values for the MBF-ratio were 92.31%, 85.19%, 87.5%, 0.962 (95% CI: 0.911–0.989, *P *< 0.001), 75%, 95.83%, and 0.731 (95% CI: 0.606–0.857, *P *< 0.001), with no significant difference (*P *= 0.1225).

**Conclusion:**

Both MBFa and the MBF-ratio exhibit excellent diagnostic performance for myocardial ischemia in patients with hemodynamically significant CAD. The MBF-ratio is more robust than MBFa for interpreting CT-MPI findings in clinical practice, which is useful for radiologists and clinicians implementing CT-MPI.

## 1 Introduction

Coronary artery disease (CAD) poses a substantial global, medical, and social burden. Coronary computed tomography angiography (CCTA) has definite diagnostic and prognostic value (IIA evidence level) in suspected CAD cases, as stipulated in the European Society of Cardiology (ESC) guidelines (1). However, CCTA offers solely anatomical information and does not present a linear correlation between the severity of coronary artery stenosis and myocardial ischemia (MI) (2). Assessing the presence and severity of myocardial ischemia is pivotal in guiding the choice between invasive or medical CAD treatment. Hence, the clinical evaluation of both coronary arteries and myocardial perfusion is essential.

Pharmacological stress dynamic computed tomography myocardial perfusion imaging (CT-MPI) is a relatively new, non-invasive functional imaging test (3). Several clinical trials have shown the usefulness of CT-MPI for diagnosing and evaluating hemodynamically significant CAD (moderate-to-severe coronary artery stenosis). Furthermore, there is a high level of expert consensus regarding its utility for determining the presence or absence of myocardial ischemia in CAD (4–6). However, the widespread adoption of CT-MPI in clinical practice faces challenges. This is partly due to the significant variation in absolute myocardial blood flow (MBFa) cutoff values—which range from 75 to 164 ml/100 ml/min—reported in studies employing diverse CT scanners and calculation algorithms (7–18). To mitigate the impact of algorithmic and individual differences on MBFa values, some studies have used the relative myocardial blood flow ratio (MBF-ratio) for diagnosing myocardial ischemia. However, the superiority of the MBF-ratio over MBFa remains inconclusive, and a standardized cutoff value has not been established (12–18). To address this challenge in applying CT-MPI to real-world settings, more clinical studies are needed.

Patients with hemodynamically significant CAD are more susceptible to myocardial ischemia and are recommended for CT-MPI (4). This study aimed to compare the diagnostic ability of two quantitative parameters—MBFa and MBF-ratio—for myocardial ischemia and explore their optimal cutoff values. To further this goal, the CT-MPI scans of patients with hemodynamically significant CAD were reviewed to acquire myocardial perfusion information.

## 2 Materials and methods

### 2.1 Study design and patients

This retrospective study was approved by the Ethics Committee of Sichuan Provincial People's Hospital (No. 2022-357). The requirement for informed consent was waived.

Adult patients with suspected or known hemodynamically significant CAD who underwent CT-MPI + CT angiography and invasive coronary angiography (ICA)/fractional flow reserve (FFR) between October 2020 and December 2023 were evaluated. The inclusion criteria were as follows: experiencing stable angina, having completed CT-MPI + CCTA, and having undergone ICA/FFR tests within 60 days and consented to participate. The exclusion criteria were as follows: myocardial infarction; postoperative coronary revascularization (e.g., stent placement or coronary artery bypass); other types of heart diseases, such as hypertrophic cardiomyopathy and hypertensive cardiomyopathy; and CT-MPI and CCTA image quality not meeting diagnostic and postprocessing requirements.

### 2.2 Preparation

CT examinations were performed using a third-generation dual-source CT scanner (SOMATOM Definition Force; Siemens Healthineers, Erlangen, Germany). The following medications were discontinued 24–48 h before the examination: beta-blockers, nitrates, calcium antagonists, dipyridamole, and aminophylline. Caffeinated beverages and foods such as coffee and cola were also not consumed within the 24 h preceding the examination. Electrocardiogram monitoring leads were attached to monitor the patients throughout the examination. Blood pressure and heart rate were assessed, and breathing exercises were conducted. Two 18-gauge cannulas were inserted into the antecubital veins.

### 2.3 Scanning procedure in CT-MPI

A calcium score scan was initiated, and CT-MPI was performed as follows. The scan range was calculated based on the calcium score images to cover the entire left ventricle (LV). Tube voltage and current were automatically adjusted using CARE Dose 4D and CARE kV according to the following parameters: reference tube voltage, 80 kV; reference tube current, 300 mAs; rotation time, 0.25 s/cycle, collimation, 48 mm × 1.2 mm; kernel of reconstruction, Qr36; and slice thickness and increment, 3 and 2.9 mm, respectively. Adenosine disodium triphosphate (ATP) was intravenously injected at a rate of 0.14 mg/kg/min using a drug pump to induce vasodilation. After 3 min of injection, 40 ml of iodinated contrast agent (Iodophor, 400 mg/ml iodine; Bracco, Milan, Italy) was injected at a rate of 5 ml/s, followed by 40 ml of normal saline at the same rate. The scan trigger delay was 5 s, and scanning was conducted using the shuttle-mode acquisition technique at the end of systole (250 ms after the R-wave) for a total scan time of 32 s. The patients were closely monitored for their safety during scanning, and ATP injection was immediately stopped upon any complication in the procedure.

### 2.4 Scanning procedure in CCTA

Nitroglycerine was administered sublingually 5–10 min after CT-MPI, and retrospective ECG-gated CCTA scanning was then performed. An automatic triggering scan of the ascending aorta was conducted with a threshold of 100 HU and a 5-s delay. We injected 40–50 ml of iodinated contrast agent (Iodophor, 400 mg/ml iodine, Bracco) at a rate of 5 ml/s, followed by saline. The following parameters were used: reference tube voltage, 100 kV; CARE Dose 4D automatic current (reference tube current, 320 mAs); rotation time, 0.25 s/cycle; collimation, 192 mm × 0.6 mm; kernel of reconstruction, Bv40; and slice thickness and increment, 0.75 and 0.5 mm, respectively.

### 2.5 Postprocessing and interpretation of CT-MPI data

Initially, the original perfusion data were transmitted to the postprocessing workstation (syngo.via, Siemens Healthineers), where the myocardial perfusion analysis software generated multiple sets of LV perfusion-related sequences. These sequences underwent respiratory-related displacement correction, noise reduction, and myocardial segmentation. The perfusion images were automatically generated, and image quality was assessed based on Likert scoring of CT-MPI as follows: 4, ≥90% segments without artifact; 3, ≥80% segments without artifact; 2, ≥70% segments without artifact; 1, ≥60% segments without artifact; scores of 2–4 were considered qualified. The qualified perfusion images and CCTA image with systolic phase were transmitted to cardiac function analysis software (version 2.0.5; Siemens Healthineers) to generate automatically a polar map of MBF and a mixed-volume rendering image of CCTA combined with MBF. Subsequently, volumes of interest (VOIs) with a minimum size of 0.5 cm^2^ were manually delineated in the lowest perfusion regions of 1–16 segments and in the highest perfusion regions of the LV. We used short-axis images with a color-coded scale for the delineation, guided by the visualization of the polar map and the mixed-volume rendering image. The VOIs were placed, allowing for only a minimal distance (1–2 mm) from the endocardial and epicardial layers to avoid contamination.

**Figure 1:**
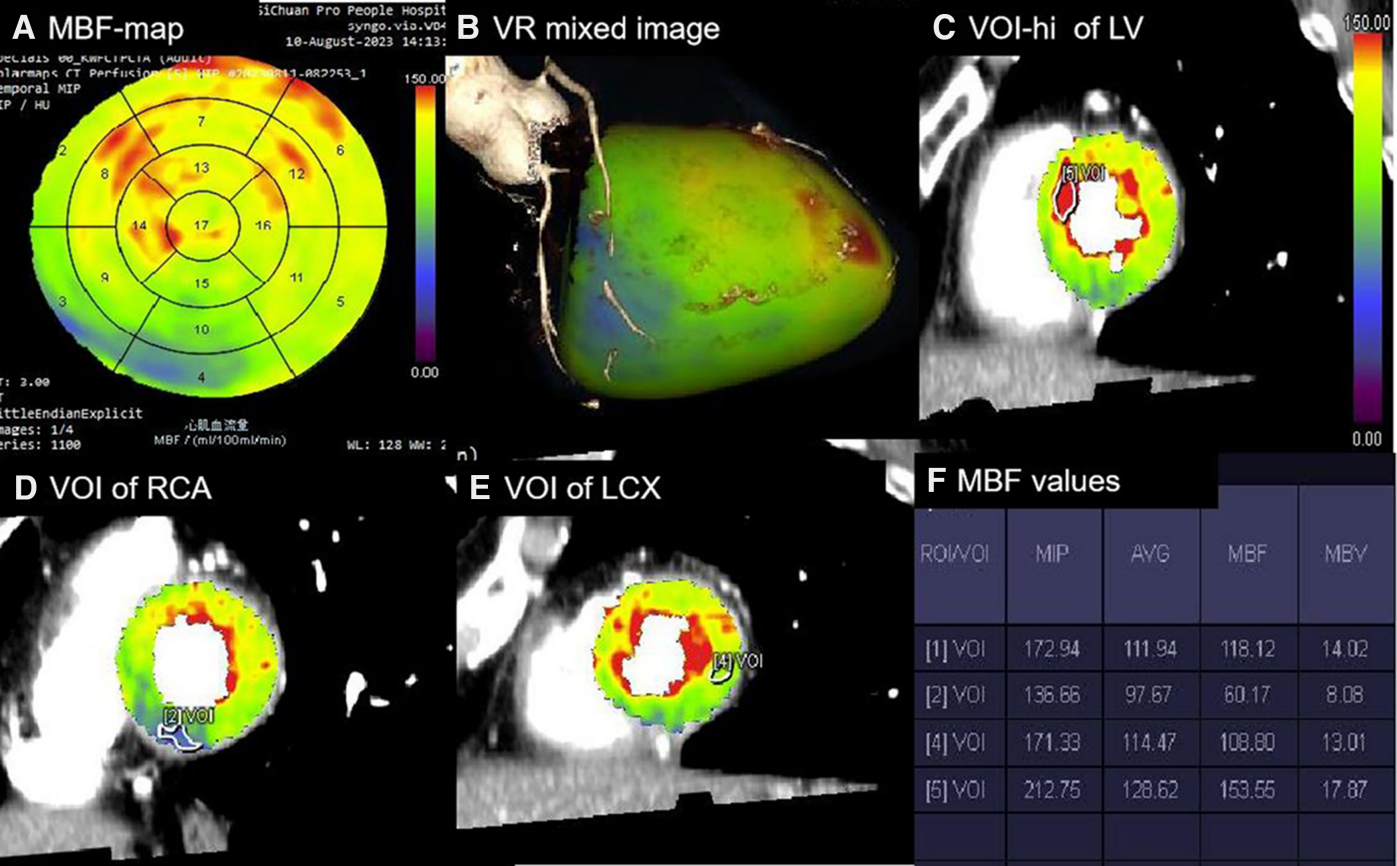
Processing of absolute and relative MBF values. (**A**) Polar map of MBF showing the MBF values in 1–17 segments with a color-coded scale. (**B**) Mixed-volume rendering image of CCTA combined with MBF showing the cardiac segments of three coronary artery territories. (**C**) VOI [5] with a minimum size of 0.5 cm^2^ manually delineated in the highest perfusion regions of the LV in the short-axis images. (**D**) VOI [2] delineated in the lowest region of the RCA territory. (**E**) VOI [4] delineated in the lowest region of the LCX territory. (**F**) Perfusion parameters automatically calculated for all VOIs [1], referring to the average perfusion of the LV. The MBF-ratio values could then be determined using the following equation: MBF-ratio = lowest MBF value/reference MBF value. RCA, right coronary artery; LCX, left circumflex artery.

The lowest MBF values within the per-vessel territory and the highest MBF (MBF-hi) and average MBF (MBF-global) values of the LV were automatically calculated. The MBF-ratio was determined using the following equation: MBF-ratio_ _= lowest MBF value/reference MBF value. In the per-patient analysis, the MBF-ratio was calculated as MBF-ratio_ _= MBF-global value/MBF-hi value; in the per-vessel analysis, it was calculated as MBF-ratio = lowest MBFa value in the per-vessel territory/MBF-hi value. VOI processing is shown in Figure 1.

### 2.6 Postprocessing and interpretation of CCTA data

For CCTA images, coronary artery analysis software (syngo.via, Siemens Healthineers) was utilized. Initially, image quality was evaluated based on Likert scoring to determine the accessibility of CCTA. Subsequently, the coronary artery calcification score was automatically calculated. Thereafter, the degrees of stenosis of the left anterior descending artery, left circumflex artery, and right coronary artery were evaluated based on the Society of Cardiovascular Computed Tomography guidelines (6). Obstructive CAD and coronary stenosis were defined as ≥50%, and ≥70%, respectively. The CT-MPI and CCTA results were evaluated by two cardiac radiologists blinded to patient data. Disagreements were resolved by consulting a third senior cardiac radiologist (with >10 years of experience). Patient information—such as age, sex, body mass index, and CAD risk factors—were obtained from the picture archiving and communication system. Changes in heart rate, blood pressure, and the dose–length product of CT-MPI were documented. The effective radiation dose (mSv) was calculated as dose − length product × k (k = 0.026 mSv mGy^−1^ cm^−1^).

### 2.7 ICA and FFR

An interventional cardiologist performed ICA or FFR within 60 days of CT-MPI + CCTA examination. ICA was performed using the standard method, and at least two views were obtained and analyzed for each major vessel. Two cardiologists visually analyzed the images without knowledge of the CCTA and CT-MPI results, referring to quantitative coronary angiography when reaching an impasse regarding the degree of stenosis. If vessels presented with intermediate stenosis (i.e., a diameter reduction of between 50% and 80% on ICA), FFR measurements were performed. FFR was performed using a sensor-tipped 0.014-in. pressure wire in these lesions during rest and maximal myocardial hyperemia induced by the venous infusion of ATP (140 μg/kg/min). FFR values ≤0.80 were considered to indicate ischemic lesions. Lesions for which FFR measurements could not be obtained were classified based on ICA; the presence and absence of myocardial ischemic lesions were defined as those with ≥80% and <50% stenosis, respectively. Vessels with intermediate stenosis but without FFR measurements were excluded. Arteries with one or more ischemic lesions were identified as vessels causing ischemia, and the most severe stenosis was considered for analysis in the same perfusion territory.

### 2.8 Statistical analysis

All statistical analyses were conducted using SPSS (version 26.0, IBM) and the MedCalc software package (MedCalc 15.2.0). Categorical variables were presented as frequencies and composition ratios (%), while continuous variables were expressed as means ± standard deviations or medians with interquartile ranges. Normally and non-normally distributed data were analyzed using the variance or *t*-test and Mann–Whitney *U*-test, respectively. The Bonferroni correction was used for multiple comparisons. Each patient and vessel were studied individually. Differences in MBFa and the MBF-ratio between the myocardial ischemic and non-ischemic groups were compared. Using ICA/FFR as a standard, receiver operating characteristic (ROC) curves were generated for each analytical method, and the area under the curve (AUC) values were compared using the DeLong test. The optimal cutoff values were determined using the Youden test. Cohen's kappa statistic was used to compare the diagnostic utility of MBFa and the MBF-ratio with that of ICA/FFR for myocardial ischemia. A bilateral *P*-value < 0.05 was considered statistically significant.

## 3 Results

### 3.1 Patient characteristics

**Figure 2:**
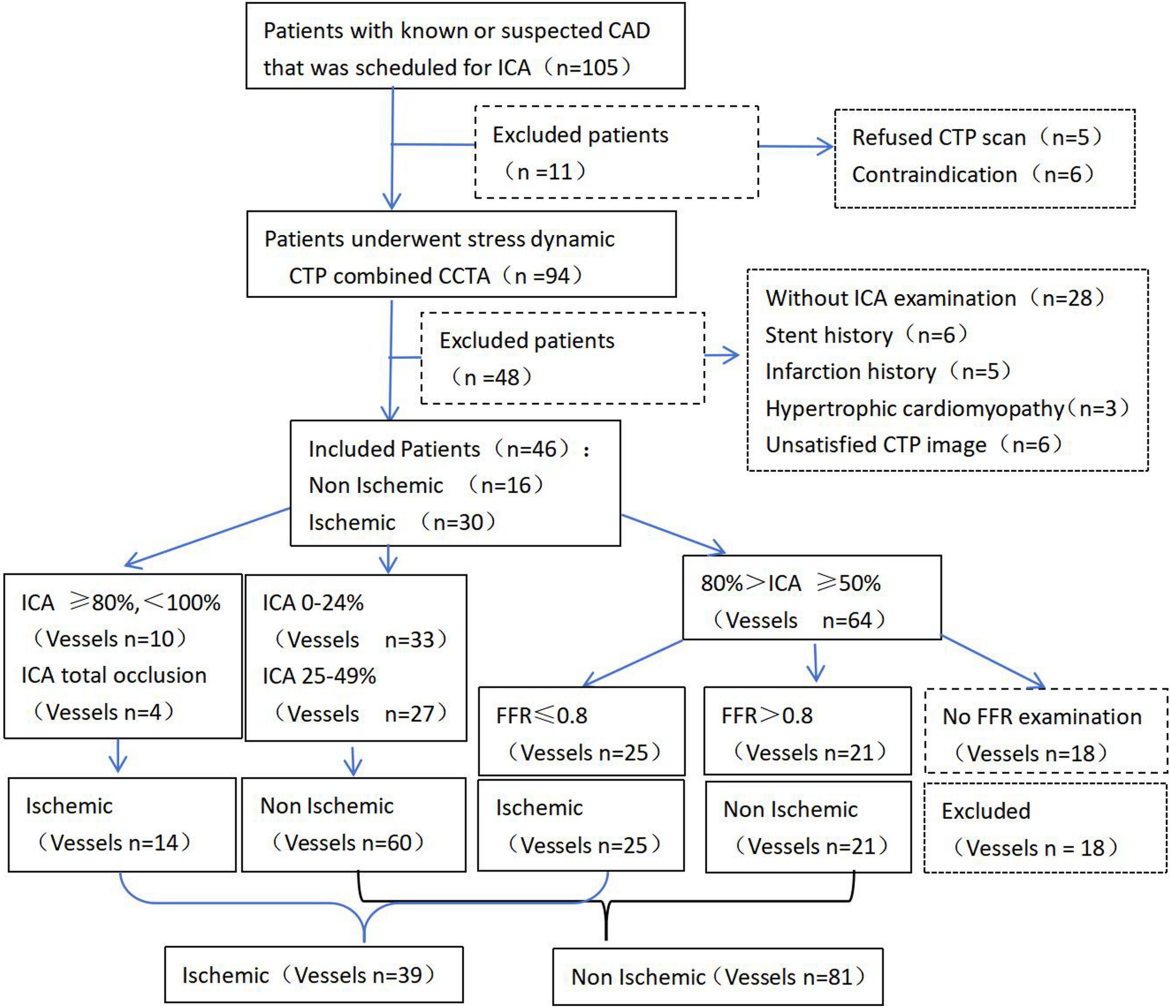
Patient inclusion flowchart.

Among the 105 patients with suspected or known hemodynamically significant CAD, 94 underwent CT-MPI combined with CCTA examinations. We excluded 48 patients due to coronary artery stenting (*n* = 6), a history of myocardial infarction (*n* = 5), hypertrophic cardiomyopathy (*n* = 3), not undergoing ICA (*n* = 28), and image quality not meeting postprocessing requirements (*n* = 6). Finally, the remaining 46 patients were evaluated; of these, 30 and 16 patients were diagnosed with the presence and absence of myocardial ischemia, respectively. Twenty-nine patients had moderate stenosis (50%–69%), while 17 patients had severe or occluded stenosis (≥70%) on CCTA. Of the 132 vessels initially evaluated, 12 vessels with 50%–80% stenosis not tested by FFR were excluded. Therefore, 120 vessels were analyzed, including 39 and 81 vessels with the presence and absence of myocardial ischemia, respectively. In total, 33, 27, 46, and 10 vessels had 0%–24%, 25%–49%, 50%–80%, and 80%–99% stenosis, respectively, and 4 vessels had chronic total occlusion on ICA. The flowchart is presented in Figure 2.

The mean age of the patients was 65.37 ± 8.25 years, and 32 patients (69.6%) were men. Compared with the non-ischemic group, the myocardial ischemic group exhibited a higher prevalence of multivessel stenosis (76.7% vs. 37.5%, *P = *0.032) and a significantly higher calcification score (*P = *0.018). Patients in the ischemic group were also older; however, there were no significant between-group differences in terms of sex or other high-risk factors for CAD. The results are presented in Table 1.

**Table 1:** Patient characteristics.

Characteristic	Total (*n* = 46)	Ischemic group (*n* = 30, 65.22%)	Non-ischemic group (*n* = 16, 34.78%)	*P*-value
Age (years)	65.37 ± 8.25	67.67 ± 7.80	61.06 ± 7.50	0.008
Sex (male)	32 (69.60%)	22 (73.30%)	10 (62.50%)	0.447
BMI	24.17 ± 2.71	24.36 ± 2.82	23.66 ± 2.12	0.350
Hypertension (yes)	24 (52.20%)	18 (60%)	6 (37.50%)	0.146
Diabetes (yes)	19 (41.30%)	15 (78.93%)	74 (25.9%)	0.185
Hyperlipidemia (yes)	21 (45.70%)	14 (46.70%)	7 (43.80%)	0.623
Family history of CAD (yes)	9 (19.60%)	7 (23.33%)	2 (12.54%)	0.850
Number of vessel lesions				0.032
Three vessels	29 (63.00%)	23 (76.70%)	6 (37.50%)	
Two vessels	10 (21.70%)	4 (13.30%)	6 (37.50%)	
One vessel	7 (15.20%)	3 (10.00%)	4 (25.00%)	
Smoking (yes)	18 (39.10%)	12 (40.00%)	6 (37.50%)	0.869
Typical angina (yes)	23 (50%)	16 (53.3%)	7 (43.75%)	0.513
Resting HR (bpm)	71.2 ± 21.25	71.04 ± 25.94	71.5 ± 7.04	0.938
Stress HR (bpm)	86.00 ± 28.15	87.54 ± 29.56	83.14 ± 26.14	0.632
Calcification score	363.01 ± 495.31	585.66 ± 106.93	136.51 ± 34.13	0.018

BMI, body mass index; HR, heart rate; bpm, beats per minute.

All participants tolerated the CT-MPI procedures well. The mean heart rate increased from 76.84 ± 12.77 bpm during rest to 90.87 ± 30.69 bpm during stress. The Likert scores for the image quality of CT-MPI were as follows: 4 for 30 patients, 3 for 12 patients, 2 for 4 patients, and 1 for 6 patients (who were excluded). The radiation exposure during CT-MPI was 297.44 ± 91.64 mGy cm, equivalent to 7.72 ± 2.38 mSv.

### 3.2 Differences between the ischemic and non-ischemic groups

**Figure 3:**
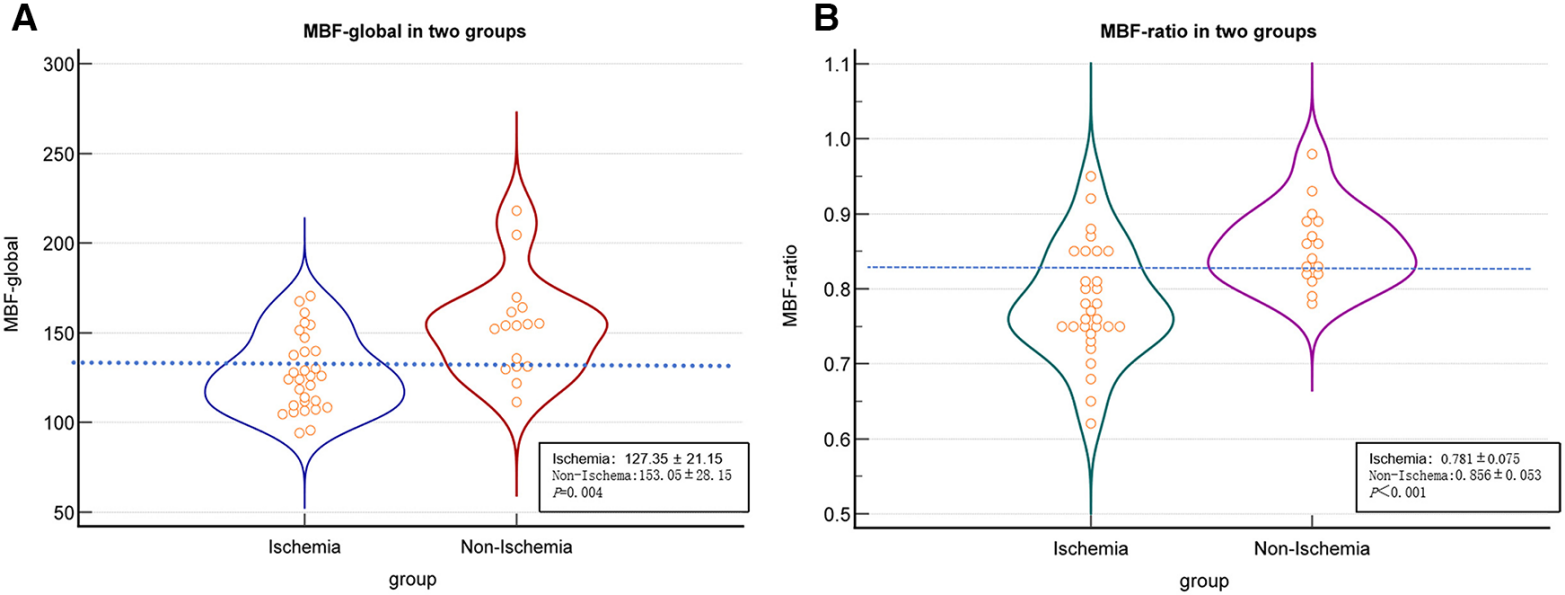
Comparison graphs of MBFa and the MBF-ratio in the ischemic and non-ischemic groups in the per-patient analysis. (**A**) Graph showing the distribution of the mean ± SD MBFa in the ischemic (blue) and non-ischemic (red) groups. The dotted line shows the optimal cutoff value (129.37 ml/100 ml/min). (**B**) Graph showing the mean ± SD MBF-ratio in the ischemic (green) and non-ischemic groups (pink). The dotted line shows the optimal cutoff value (0.81).

**Figure 4:**
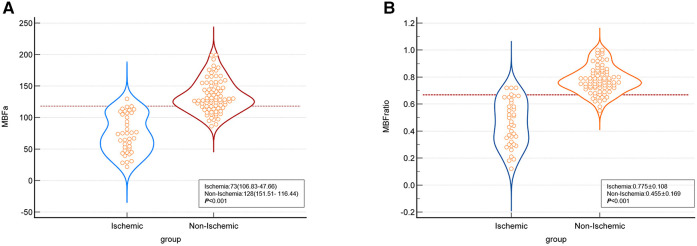
Comparison graphs of MBFa and the MBF-ratio in the ischemic and non-ischemic groups in the per-vessel analysis. (**A**) Graph showing the distribution of the mean ± SD MBFa in the ischemic (blue) and non-ischemic (red) groups. The dotted line shows the optimal cutoff value (117.71 ml/100 ml/min). (**B**) Graph showing the distribution of the mean ± SD MBF-ratio in the ischemic (blue) and non-ischemic groups (orange). The dotted line shows the optimal cutoff value (0.67).

In the per-patient analysis, both the MBF-global and MBF-ratio were significantly lower in the ischemic than in the non-ischemic group (127.35 ± 21.15 vs. 153.05 ± 28.15, *P = *0.004, and 0.781 ± 0.075 vs. 0.856 ± 0.053, *P *< 0.001, respectively). The results are presented in Figure 3 and Table 2. Similar results were obtained in the per-vessel analysis. Both MBFa and the MBF-ratio were significantly lower in the ischemic group than in the non-ischemic group (73,47.66–106.83 vs. 128,116.44–151.51, *P *< 0.001, and 0.455 ± 0.169 vs. 0.775 ± 0.108, *P *< 0.001, respectively). The degree of coronary artery stenosis was significantly more severe in the ischemic group (*P *< 0.001); approximately half of the patients in the non-ischemic group had only moderate stenosis. The results are presented in Figure 4 and Table 3.

**Table 2:** MBF parameters in the per-patient analysis.

	Total (*n* = 46)	Ischemic group (*n* = 30, 65.22%)	Non-ischemic group (*n* = 16, 34.78%)	*P*-value
Highest MBF value (ml/100 ml/min)	168.70 ± 27.70	163.38 ± 25.30	178.66 ± 30.04	0.095
MBF-global value (ml/100 ml/min)	136.29 ± 26.57	127.35 ± 21.15	153.05 ± 28.15	0.004
MBF-ratio value	0.807 ± 0.076	0.781 ± 0.075	0.856 ± 0.053	<0.001

**Table 3:** Comparison of quantitative parameters between the ischemic and non-ischemic groups in the per-vessel analysis.

	Total vessels (*n* = 120)	Ischemic vessels (*n* = 39, 32.5%)	Non-ischemic vessels (*n* = 81, 67.5%)	*P*-value
Diameter narrowing of coronary lesions by ICA (n, %)				<0.001
0: Negative (0%)	18 (15%)	0 (0%)	18 (22.2%)	
1: Minimal stenosis (1–24%)	15 (12.5%)	0 (0%)	15 (18.52%)	
2: Mild (25–49%)	27 (22.5%)	0 (0%)	27 (33.3%)	
3: Moderate (50–79%)	46 (38.33%)	25 (64.1%)	21 (25.93%)	
4: Severe (80–99%)	10 (8.33%)	10 (25.64%)	0 (0%)	
5: Occluded (100%)	4 (3.33%）	4 (10.26%)	0 (0%)	
Diameter narrowing of coronary lesions by CCTA (*n*, %)				<0.001
0: None	18 (15%)	0 (0%)	18 (22.2%)	
1: Minimal stenosis (1–24%)	14 (11.67%)	0 (0%)	14 (17.28%)	
2: Mild (25–49%)	24 (20%)	0 (0%)	24 (29.63%)	
3: Moderate (50–69%)	45 (37.5%)	22 (56.4%)	23 (28.40%)	
4: Severe (70–99%)	15 (12.5%)	13 (33.33%)	2 (2.47%)	
5: Occluded	4 (3.33%）	4 (10.26%)	0 (0%)	
Highest MBF (ml/100 ml/min)	164.5 (149–185)	160 (148–179)	165 (150.34–192)	0.079
MBF-ratio	0.722 (0.58–0.80)	0.775 ± 0.108	0.46 ± 0.17	<0.001
MBFa (ml/100 ml/min)	117.83 (95.05–138.97)	73 (47.66–106.83)	128 (116.44–151.51)	<0.001

### 3.3 Diagnostic efficacy of MBFa and MBF-ratio

**Figure 5:**
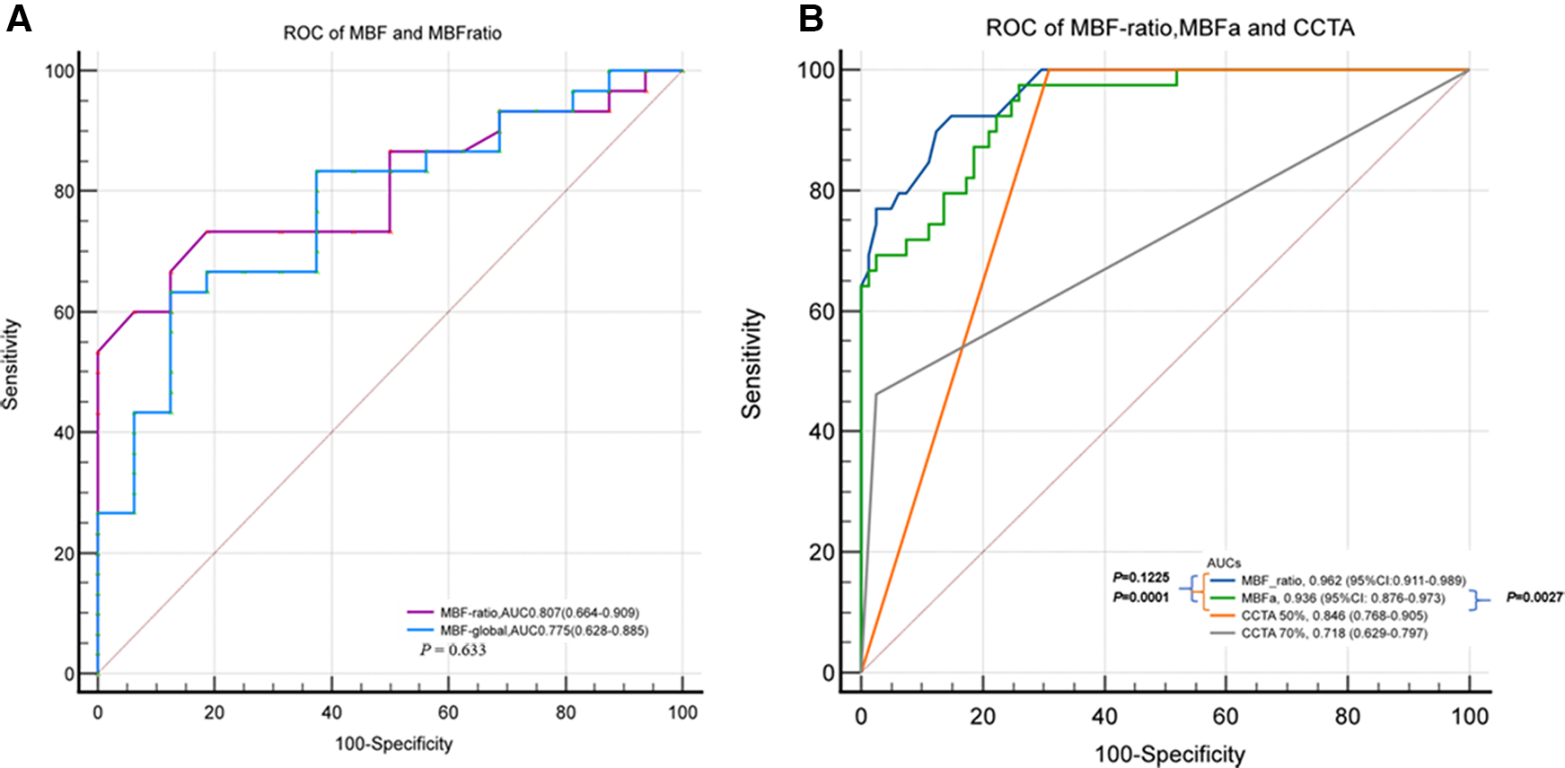
ROC curves of MBFa and the MBF-ratio for detecting MI. (**A**) ROC curves showing the AUCs of MBFa (0.705) and the MBF-ratio (0.807) for detecting MI (0.705 vs. 0.807, *P = *0.633) in the per-patient analysis. (**B**) ROC curves showing that the AUCs of MBFa and the MBF-ratio are higher than that of CCTA for detecting MI with ≥50% and ≥70% stenosis (0.936 and 0.962 vs. 0.846 and 0.718, *P* < 0.05, respectively) in the per-vessel analysis.

In the per-patient analysis, the MBF-ratio had a higher AUC than the MBF-global for the diagnosis of myocardial ischemia; however, the difference was not significant [0.807, 95% confidence interval (CI): 0.664–0.909, vs. 0.775, 95% CI: 0.628–0.885, *P = *0.633]. Similarly, the MBF-ratio had a higher AUC than MBFa in the per-vessel analysis; the difference was still not significant [0.962 (95% CI: 0.911–0.989) vs. 0.936 (95% CI: 0.876–0.973), *P = *0.1225]. The AUCs of ≥50% and ≥70% stenosis on CCTA were 0.846 (95% CI: 0.768–0.905) and 0.718 (95% CI: 0.629–0.797), respectively. Both MBFa and the MBF-ratio had higher AUCs than CCTA (*P *< 0.005). The results are presented in Figure 5 and Table 4.

**Table 4:** Diagnostic efficacy of MBFa and the MBF-ratio for myocardial ischemia.

	Sen (%)	Spe (%)	Acc (%)	PPV % (%)	NPV (%,	AUC (95% CI)	*P*-value	Cutoff value	Youden index	Kappa value (95% CI)
Per- patient										
MBFa	63.33 (19/30)	87.50 (14/16)	71.74 (33/46)	90.48 (19/21)	56.00 (14/25)	0.775 (0.628–0.885)	<0.001	129.37	0.508	
MBF-ratio	73.33 (22/30)	81.25 (13/16)	76.09 (35/46)	88.00 (22/25)	61.90 (13/21)	0.807 (0.664–0.909)	<0.001	0.81	0.546	
Per-vessel										
MBFa	97.44 (38/39)	74.07 (60/81)	81.66 (98/120)	63.33 (38/59)	98.36 (60/61)	0.936 (0.876–0.973)	<0.001	117.71	0.715	0.631 (0.500–0.762, *P *< 0.001)
MBF-ratio	92.31 (36/39)	85.19 (69/81)	87.50 (105/120)	75.00 (36/48)	95.83 (69/72)	0.962 (0.911–0.989)	<0.001	0.67	0.775	0.731 (0.606–0.857, *P *< 0.001)
CCTA (50%)	100.00 (39/39)	69.14 (56/81)	79.17 (95/120)	60.94 (25/64)	100 (56/56)	0.846 (0.768–0.905)	<0.001	50%	0.691	
CCTA (70%)	46.15 (17/39)	97.53 (79/81)	79.47 (96/120)	89.47 (17/19)	78.22 (79/101)	0.718 (0.629–0.797)	<0.001	70%	0.437	

The AUCs were compared using the DeLong test: CCTA (50%) vs. MBF-ratio, *P* = 0.0001; CCTA (50%) vs. MBFa, *P* = 0.0027; CCTA (70%) vs. MBF-ratio, *P* < 0.0001; CCTA (70%) vs. MBFa, *P* < 0.0001; MBF-ratio vs. MBFa, *P* = 0.1225; CCTA (50%) vs. CCTA (70%), *P* = 0.0076.

Sen, sensitivity; Spe, specificity, Acc, accuracy; NPV, negative predictive value; PPV, positive predictive value.

**Figure 6:**
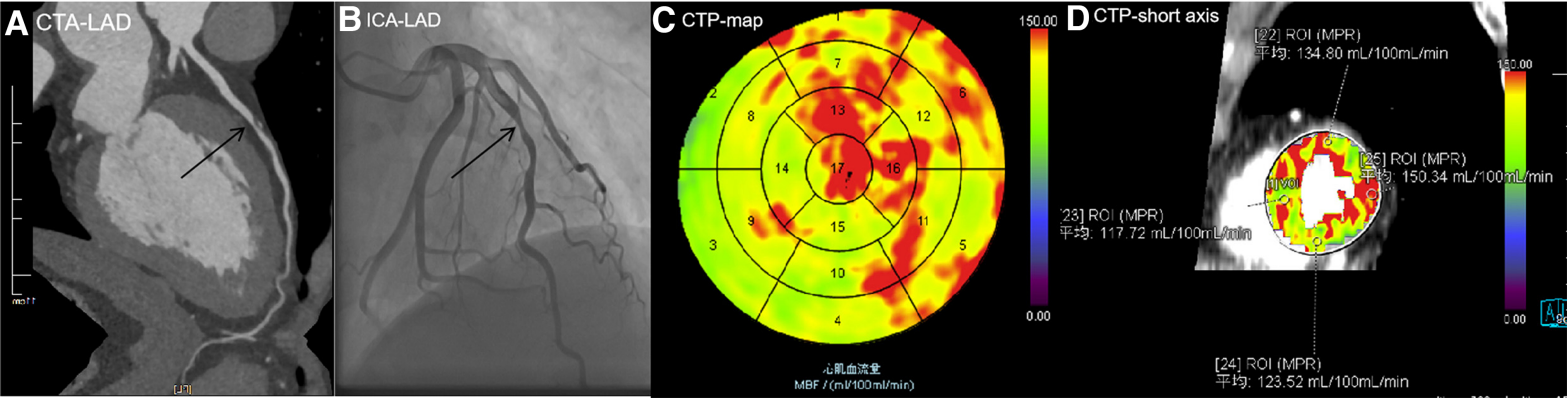
A 56-year-old man with type 2 diabetes for 1 year presented with chest pain. (**A**) CCTA image showing atherosclerotic plaques in the middle of the LAD with severe stenosis of 80%, indicating MI. (**B**) ICA showing 75% stenosis in the middle of the LAD, with an FFR value of 0.85. No revascularization therapy was performed. (**C**) CTP-MBF map image showing that the LAD territory (segment 14) was relatively lower than other areas. The MBF-global value was 131.15 ml/100 ml/min. (**D**) MBF short-axis image showing that all MBFa values were higher than the cutoff value (117.71 ml/100 ml/min), the lowest MBF-ratio value in segment 14 was 0.783 (117.72/150.34), and there was no evidence of MI. In this patient, moderate stenosis of the coronary artery did not cause MI, and MBFa and MBF-ratio values were consistent with FFR. LAD, left anterior descending artery; CTP, computed tomography perfusion.

**Figure 7:**
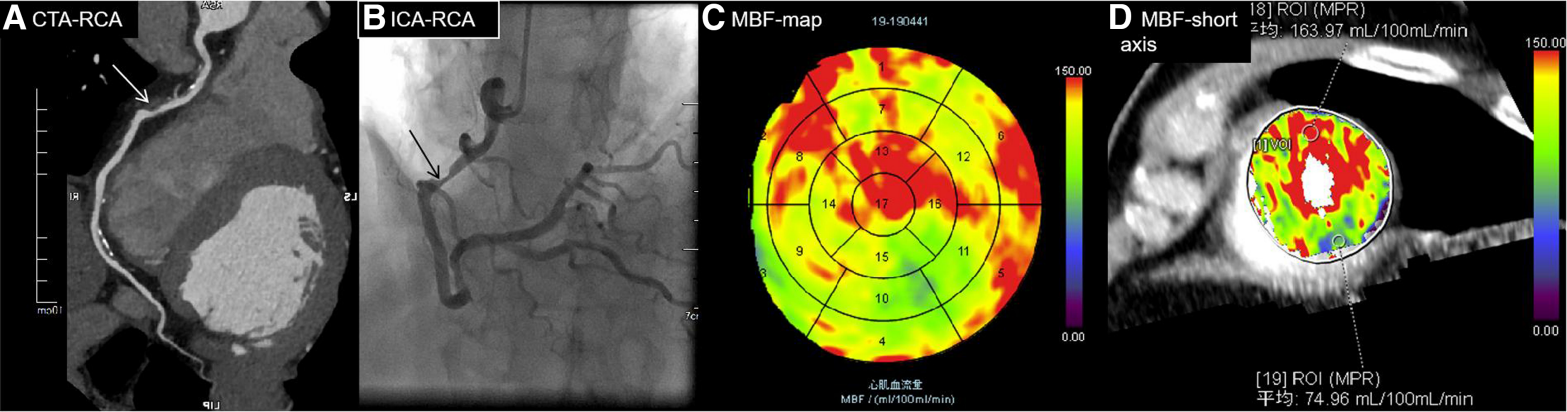
A 70-year-old man diagnosed with hypertension and diabetes for 7 years presented with chest pain. (**A**) CTA showing moderate stenosis of 75% in the middle RCA (white arrow), indicating MI. (**B**) ICA showing severe stenosis of 80% in the middle of the RCA (black arrow), with an FFR value of 0.7, indicating MI. (**C**) MBF map image showing decreased perfusion in the RCA territory (blue and green areas in segments 10, 11, and 15). (**D**) MBF short-axis image showing decreased perfusion in the inferior wall of the left ventricle, specifically at region of interest (ROI) 19. The MBFa value is 74.96 ml/100 ml/min, and the MBF-ratio is 0.46 (74.96/163.97) (highest MBF, ROI 18). The MBFa and MBF-ratio values were consistent with FFR.RCA, right coronary artery.

The optimal cutoff value for the MBF-ratio was determined to be 0.67, with a sensitivity (high), specificity (good), accuracy, positive predictive value (PPV), and negative predictive value (NPV) of 92.31%, 85.19%, 87.5%, 75%, and 95.83%, respectively (*P *< 0.001). The MBF-ratio demonstrated strong consistency with ICA/FFR, exhibiting a kappa value of 0.731 (95% CI: 0.606–0.857, *P *< 0.001). For MBFa, the optimal cutoff value was 117.71 ml/100 ml/min, with a sensitivity (high), specificity (moderate), accuracy, PPV, and NPV of 97.44%, 74.07%, 81.66%, 63.33%, and 98.36%, respectively. The MBFa value was also strongly consistent with ICA/FFR, with a kappa value of 0.631 (95% CI: 0.500–0.762, *P *< 0.001). Stenosis ≥50% on CCTA demonstrated a sensitivity (high), specificity (moderate), accuracy, PPV, and NPV of 100%, 69.14%, 79.17%, 60.94%, and 100%, respectively; stenosis ≥70% on CCTA demonstrated higher specificity (97.53% vs. 69.14%) but lower sensitivity (46.15% vs. 100%) for the detection of hemodynamically significant CAD, and the accuracy, PPV, and NPV were 79.47%, 89.47%, and 78.22%, respectively. The results are presented in Table 4. Two representative cases are presented in Figures 6, 7.

## 4 Discussion

### 4.1 Main findings

This study shows that both MBFa and the MBF-ratio have excellent diagnostic efficacy for myocardial ischemia; however, the MBF-ratio has more balanced diagnostic sensitivity and specificity and is more robust and reliable. Furthermore, in the per-vessel analysis, the optimal cutoff values for MBFa and the MBF-ratio for diagnosing myocardial ischemia were 117.71 ml/100 ml/min and 0.67, respectively.

### 4.2 Diagnostic performance of CT-MPI compared with CCTA

The ischemic group included more patients with multivessel lesions and severe calcification, and the degree of vessel stenosis was more severe than in the non-ischemic group. Conversely, approximately half of the vessels had moderate stenosis in the non-ischemic group. This confirmed that the degree of stenosis was not completely proportional to myocardial ischemia in moderately stenotic vessels. Thus, further functional examinations are necessary for vessels with moderate stenosis. CT-MPI is helpful for the diagnosis of myocardial ischemia. Compared with CCTA alone, CT-MPI combined with CCTA has demonstrated significantly better accuracy for myocardial ischemia diagnosis (7–9, 16, 17). Kitagawa et al. (17) found that, compared with CCTA alone (≥50% stenosis), CT-MPI combined with CCTA had significantly higher diagnostic specificity (36% vs. 75%, *P *< 0.001) and accuracy (64% vs. 74%, *P *< 0.001), especially in the diagnosis of moderate stenosis (44% vs. 71%, *P *< 0.001). Our study demonstrated similar results: compared with CCTA (≥50% stenosis), CT-MPI using the MBF-ratio had significantly higher diagnostic specificity (69.14% vs. 85.1%, *P *< 0.001) and accuracy (79.17% vs. 87.5%, *P *< 0.001). Furthermore, MBFa had better diagnostic accuracy (79.17% vs. 81.6%, *P *< 0.001) in the per-vessel analysis. Compared with CCTA (≥70% stenosis), CT-MPI using both the MBF-ratio and MBFa had significantly higher sensitivity (46.15% vs. 97.55% and 92.31%, *P *< 0.001); this is similar to the results of a previous study (16). Furthermore, CT-MPI also demonstrated a better balance between sensitivity and specificity than CCTA. Yu et al. (19) also reported that, compared with CCTA alone, CCTA combined with CT-MPI is associated with a lower incidence of ICAs without vascular revascularization; thus, unnecessary ICA examinations can be avoided, especially in patients with moderate stenosis.

### 4.3 Diagnostic efficacy and optimal cutoff value of MBFa

MBFa values were significantly lower in the ischemic group than in the non-ischemic group in both per-patient and per-vessel analyses. In the per-vessel analysis, the MBFa value had an excellent AUC value of 0.936 (95% CI: 0.876–0.973), a high sensitivity of 97.44%, and a moderate specificity of 74.07%. It also had good consistency with the standard (0.631, 95% CI: 0.50–0.762). These results were consistent with those of previous studies. In a meta-analysis, the AUC of CT-MPI reached 0.911, and the sensitivity and specificity were 85% and 81%, respectively (20). The optimal cutoff value for MBFa varies substantially from 75 to 164 ml/100 ml/min (12–18); in our study, it was 117.71 ml/100 ml/min, consistent with the results of studies that used the same CT scanner and standards (20). It was also consistent with the lower limit of the normal range found by a Chinese study (116 ml/100 ml/min) (21). The main reason for the wide variation in the optimal cutoff value for MBFa may be due to differences in the degree of myocardial ischemia among patients. For example, in studies (7, 12) that included patients with myocardial ischemia and a history of revascularization, the optimal cutoff value for MBFa was significantly lower. By contrast, the optimal cutoff value was higher in studies that involved a relatively lower proportion of lesions in three vessels and severe stenosis (13, 16).

### 4.4 Diagnostic efficacy and optimal cutoff value for the MBF-ratio

Given the substantial variation in the optimal cutoff value for MBFa in previous studies, we tried to use the MBF-ratio; still, the results were inconsistent. Wichmann et al. (12) and Kono et al. (18) reported that the AUCs of the MBF-ratio were significantly higher than those of MBFa (0.925 vs. 0.882, *P *= 0.0022, and 0.85 vs. 0.75, *P *= 0.02, respectively). By contrast, Yi et al. (14) reported a significantly higher AUC value of MBFa (0.955 vs. 0.906, *P *= 0.02). Several studies (13, 15, 16) also reported higher AUCs for the MBF-ratio compared with those for MBFa for detecting ischemia. Specifically, the AUCs were 0.90 vs. 0.87, 0.956 vs. 0.942, and 0.82 vs. 0.79, respectively, in these studies; however, the difference was not statistically significant (*P *> 0.05). The current study showed that the MBF-ratio was significantly lower in the ischemic than in the non-ischemic group in both per-patient (*P = *0.02) and per-vessel analysis (*P *< 0.01). The AUC value of the MBF-ratio in the per-vessel analysis was higher than that of the MBFa, albeit without a significant difference (0.936 vs. 0.962, *P = *0.1225). However, the MBF-ratio showed a trend of a better balance between sensitivity (92.31%) and specificity (85.19%) than MBFa (sensitivity, 97.44%; specificity, 74.07%). In addition, the MBF-ratio exhibited better consistency with the diagnostic standards for functional ischemia (ICA ≥80% or FFR ≤0.8). Collectively, these results indicated that the MBF-ratio demonstrated equal or superior diagnostic efficacy compared with the MBFa for myocardial ischemia diagnosis.

The optimal cutoff values for the MBF-ratio range from 0.675 to 0.85 (12–18), with an optimal value of 0.67 in the current study. These values align closely with the pressure drop for myocardial ischemia diagnosis by FFR (0.75–0.8). The highest MBF can be selected as a reference to simulate the maximum initial normal state of the coronary artery. Overall, compared with MBFa, the MBF-ratio exhibits a relatively smaller range of variation, making it more robust and reliable in real-world clinical applications. One possible reason for the different performance of the MBF-ratio is the difference in study design (12–18, 22), particularly the different denominators (remote myocardium of reference) used as reference MBF values. These included the highest segmental MBF value without artifacts (12, 18) and the highest automatic segmental value of the endocardium (14), the third quartile of the average segmental value (13), and the average MBF value of the LV (23, 24) and all the myocardial segments supplied by vessels with <30% stenosis (14). These MBF values were all used as reference MBF values for calculating the MBF-ratio. Theoretically, using the MBF-hi as a reference MBF value should yield lower cutoff values than other MBF values. Using the third quartile or average MBF value as a reference may result in an underestimation of the identified reference value to some extent, especially in patients with multivessel stenosis. This results in an amplification of relative proportions and an increase in false-negative results; thus, the MBF-hi may be a better reference to simulate the maximum initial normal state of the coronary artery (22). Another reason for different performance is a different numerator (endocardial, epicardial, or transmural perfusion). Endocardial analysis makes perfusion defects more apparent than transmural and epicardial assessments (18). For predicting ischemia, the MBF-ratio of the endocardial myocardium layer as a numerator performs better than the ratio from the transmural layer (13, 22). In our study, we drew VOIs in the transmural layers to avoid artifacts related to myocardial displacement of the LV.

Given the different perfusion between the endocardium and epicardia, the transmural perfusion ratio (TPR) and endocardial-to-epicardial MBF-ratio are also used to detect myocardial ischemia. The TPR has a lower AUC than a visual analysis of myocardial perfusion (0.759 vs. 0.877, *P *= 0.002) (25) while exhibiting comparable performance to that of the absolute MBF value (26). In previous studies, the AUC of the TPR was significantly higher than that of the MBF for the detection of flow-limiting CAD (0.833 vs. 0.711, *P *= 0.0273) (27). However, the diagnostic utility of the TPR for detecting myocardial ischemia still needs to be further explored.

### 4.5 Radiation dose

The disadvantages of dynamic CT-MPI include the radiation dose. However, this study used a third-generation dual-source CT system with radiation exposure of 7.72 ± 2.38 mSv, a nearly 30% reduction in dose from previous studies utilizing a second-generation dual-source CT system wherein the radiation exposure ranged from 588 to 757 mGy cm, i.e., 8.23–10.6 mSv (3, 5). One study attempted to reduce the radiation dose of CT perfusion to 3.8 ± 1.4 mSv by lowering the tube voltage to 70 kV (22).

### 4.6 Limitations

First, this was a retrospective, single-center, small-sample study; thus, the findings may apply only to similar examination protocols using third-generation dual-source CT. Second, diagnosing myocardial ischemia requires reference to the normal range in healthy individuals. Third, MBF was measured semiautomatically on the axial images, which may have resulted in interobserver differences and long postprocessing times. Fourth, FFR was performed using a visual analysis of ICA with 50%–80% stenosis, which may have influenced the results of the study. Finally, the study included patients with non-ischemic and chronic occlusion, which may have led to an overevaluation of the performance of CT-MPI. Multicenter prospective trials are needed to investigate further automated methods for measuring the relative MBF. The diffusion and clinical use of automatic software offering standardization of CT-MPI quantitative data will help define the best approach in different clinical settings in the future. The application of new techniques, such as the use of deep learning to remove motion artifacts of the LV, will also help improve the effectiveness of quantitative parameters for diagnosing myocardial ischemia.

### 4.7 Conclusions

In conclusion, stress dynamic CT-MPI offers high spatial resolution, complete left ventricle coverage, and the ability to correlate perfusion abnormalities with coronary CCTA results, thus seamlessly integrating anatomy and function. Importantly, the MBFa and MBF-ratio have excellent diagnostic efficacy in diagnosing myocardial ischemia, and the relative MBF-ratio has a more balanced diagnostic sensitivity and specificity. The result is particularly useful for radiologists and clinicians, addressing a common challenge in applying CT-MPI in real-world settings and thus potentially impacting the accurate diagnosis and management of CAD.

## Data Availability

The original contributions presented in the study are included in the article/Supplementary Material, further inquiries can be directed to the corresponding authors.
